# A comparative study on the effect of blood collection tubes on stress oxidative markers

**DOI:** 10.1371/journal.pone.0266567

**Published:** 2022-04-06

**Authors:** Alireza Bastin, Saba Fooladi, Amir Hossein Doustimotlagh, Sina Vakili, Amir Hashem Aminizadeh, Sanaz Faramarz, Hamidreza Shiri, Mohammad Hadi Nematollahi

**Affiliations:** 1 Department of Biochemistry, Faculty of Medicine, Bushehr University of Medical Sciences, Bushehr, Iran; 2 Student Research Committee, Kerman University of Medical Sciences, Kerman, Iran; 3 Department of Clinical Biochemistry Faculty of Medicine, Yasuj University of Medical Sciences, Yasuj, Iran; 4 Infertility Research Center, Shiraz University of Medical Sciences, Shiraz, Iran; 5 Physiology Research Center, Kerman University of Medical Sciences, Kerman, Iran; 6 Herbal and Traditional Research Center, Kerman University of Medical Sciences, Kerman, Iran; 7 Department of Biochemistry, Faculty of Medicine, Kerman University of Medical Sciences, Kerman, Iran; Tribhuvan University, NEPAL

## Abstract

Oxidative stress has a major role in disease pathogenesis. However, limited studies have investigated the effect of various sample collection tubes on oxidative biomarkers. The present study aimed to evaluate the effect of different collection tubes on the variation of malondialdehyde (MDA), nitric oxide (NO), total thiol (t-SH), and ferric reducing ability of plasma (FRAP) levels. A total of 35 individuals participated in this study and each collected sample was separated into three different tubes: glass tubes (GTs), plain plastic tubes (PTs), and gel separator tubes (GSTs). The results of PTs and GSTs were compared to those of GTs as the reference tube. The comparison between the means of biomarkers in various tubes indicated that there was no significant difference in MDA results between tubes. In contrast, t-SH and NO content were significantly decreased in GSTs and PTs compared to GTs. However, the Bland-Altman analysis showed an acceptable concordance for the mentioned analytes and the statistically significant differences were not clinically significant for NO, MDA, and t-SH antioxidant parameters. Moreover, the FRAP level was considerably lower in GSTs compared to GTs. Nevertheless, the Bland-Altman analysis showed a high bias percentage for the FRAP assay when using PTs and GSTs. According to the present results, it can be concluded that switching to plastic blood collection tubes or serum separation tubes could influence the FRAP results. However, there was no interference for the interpretation of other antioxidant assays in different types of collection tubes. Hence, it is suggested to use GTs for total antioxidant capacity evaluations, especially the FRAP assay.

## Introduction

Blood collection is a critical step in pre-analytical laboratory testing and plays an integral role in the accuracy of the results. Previously, the most prevalent tubes used for blood collection were glass ones. However, they were replaced by plastic tubes which are usually made up of materials such as polyesters, polyolefins, polysiloxane, polyacrylonitrile, polyacrylic, polyvinyl chloride, polytetrafluoroethylene, and polystyrene [1, 2]. Since these substances are hydrophobic, the activation of the coagulation process is postponed in these tubes, and additives, e.g., polymer gels, clot activators, and surfactants are employed to resolve this issue [1]. Clot activators are composed of inorganic silicate or substances such as thromboplastin, thrombin, and ellagic acid. However, it is likely that these substances may not sediment with the clot after centrifugation, stay in serum, and cause interference with various assays [2–5]. Moreover, surfactants are usually fabricated from polymers that are soluble in water and it has been demonstrated that some biochemical factors such as thyroid hormones are altered in the presence of surfactants [2, 6–9]. Nevertheless, plastic tubes are still widely employed in laboratories owing to their ease of use, ability to increase serum volume, and reduced risk of contamination [10–12]. Despite these advantages, there are controversies around the precision of laboratory tests on samples gathered in different kinds of tubes, and several studies have proposed alternations in the concentration of biochemical analytes in plastic tubes versus glass ones. It has been established that tubes containing separator gels are not only able to absorb hydrophobic compounds such as specific drugs but also release an oily film to serum under extreme temperature because of their instability, which clogs the instrument probe in the analytical step [13]. Consequently, it has been suggested that serum/plasma separator tubes may have a slight analytical effect on several assays including hormonal, drug monitoring, and immunoassay tests because total triiodothyronine (TT3) and total thyroxine (TT4) concentrations are increased in plain red tubes, serum separator tubes, and rapid serum tubes compared to glass tubes (GTs) [8, 10, 14]. Moreover, the concentration of some drugs such as carbamazepine is significantly elevated in plastic tubes after 24 h [14]. Despite considering a wide spectrum of laboratory assays, we found that the effect of various collection tubes on stability and the accuracy of measured metabolites produced during oxidative stress has never been studied.

Oxidative stress status caused by the impairment of oxidant/antioxidant equilibrium is shown to have a major role in disease pathogenesis and has been studied in a wide spectrum of disciplines such as chemistry, biology, biochemistry, and physiology owing to its importance in cell survival and maintaining the crucial functions of the cell [15]. During oxidative stress, reactive species (RS) trigger modifications in biomolecules including DNA, lipids, and proteins [16]. Nitric oxide (NO) is one of these products, which is generated by intact endothelium and plays a role in oxidative stress alongside other reactive oxygen species (ROS) [17]. In addition, lipid peroxidation of polyunsaturated fatty acids (PUFAs) in membranes is usually evaluated by the formation of malondialdehyde (MDA), which is one of the principal breakdown products of the endoperoxidase activity in various diseases [18]. In contrast, a number of assays have been developed to assess the total antioxidant power of plasma. For instance, the measurement of the ferric reducing ability of plasma (FRAP) and total thiol (t-SH) is introduced to evaluate the antioxidant status [19, 20]. Several studies have indicated that investigating the profile of protein modifications (thiol groups) caused by oxidative stress would be a favorable approach for facilitating the diagnosis of pathological conditions and several disorders including inflammatory responses, atherosclerosis, and neurodegenerative disorders. As a result, studying oxidative stress biomarkers is crucial in determining the oxidative stress status in individuals to help the identification of pathological conditions [21–24]. Therefore, the collection of blood in an appropriate tube has great importance in the precision and reliability of the test results. However, many laboratories have made little effort to evaluate the quality of blood collection tubes and monitor their performance.

To the best of the authors’ knowledge, the impact of using different blood collection tubes on the variations of oxidative stress metabolites is not well-known and only a few authors have sporadically addressed this issue. A study investigating the alternations of myeloperoxidase collected in various tubes revealed that heparin plasma tubes show higher myeloperoxidase concentrations than EDTA or citrate tubes [25]. However, this study focused on the effects of two different anticoagulants not the type of tube itself. Hence, the present study aimed to investigate the impact of GTs, plain plastic tubes (PTs), and gel separator tubes (GSTs) on the oxidative (MDA and NO) and antioxidative markers including t-SH levels as well as total antioxidant capacity, which is measured by the FRAP assay, in order to suggest an appropriate blood collection tube to minimize the spontaneous effects on antioxidant markers.

## Materials and methods

### Sample collection

Three types of blood collection tubes were examined in this study, including GT (non-silicone coated glass), PT (plastic with clot activator), and GST (gel separator with clot activator). All tubes were 14 x 100 mm blood collection tubes with a sterile interior. In the present study, GTs were considered the control tubes since they have been the conventional serum collecting tubes over the past few decades and contain neither surfactants for the coverage of internal tube surface nor clot activators and separator gels [8]. The project was approved by the ethics committee of Kerman University of Medical Sciences (Ethical Approval Code: IR.KMU.REC.1398.177). Written informed consent, as a crucial requirement for providing the participants with the details of the proposed trial, was obtained from all the contributors according to the principles of the Declaration of Helsinki. Afterward, 10 mL of venous blood was drawn from 35 healthy volunteers aged between 18 and 50 years after 10 h of fasting. The blood samples were separated into the previously mentioned tubes and inverted eight times to ensure the proper mixing of blood and additives. The samples were incubated for 30 min at room temperature and serum was separated by centrifugation at 800g for 8 min. All the serum samples were stored at -70°C until analysis.

### Oxidative stress markers

#### Determination of MDA

Serum MDA level was determined according to the method introduced by Buege and Aust (1978) as described in detail in our previous study [26]. The color produced as a result of thiobarbituric acid (TBA) and MDA reaction was assessed at 532 nm. Consequently, a molar absorption coefficient of 1.56 × 105 M^−1^ cm^−1^ was employed to estimate the MDA level and the results are expressed as μmol/L.

#### Determination of the NO metabolite

The Griess method was applied to measure the NO content. For this purpose, the serum samples were deproteinized using acetonitrile followed by 30 min incubation at 37°C after the addition of 0.1 mL of the Griess reagent. Subsequently, the samples’ absorbance was determined at 546 nm and the NO level was evaluated using a standard curve confirmed by 0–50 μmol/L sodium nitrite. The results are expressed as μmol/L [27].

#### Determination of t-SH

To calculate the t-SH content in the cell lysate, the spectrophotometric procedure was performed. In this assay, the 5,5′-dithiobis (2-nitrobenzoic acid) (DTNB) reagent was used, which produces a yellow complex when it reacts with thiol groups and has the maximum absorption at 412 nm [28]. The t-SH amount was determined using the molar absorption coefficient of 13,600 M^−1^ cm^−1^.

#### Determination of FRAP levels

The FRAP assay was carried out based on the Benzie and Strain method in which the reduction ability of ferric (III) to ferrous (II) ion at a low pH is evaluated [20]. Concisely, the addition of cellular supernatant to the FRAP reagent creates a blue color, which is spectrophotometrically assessed at 593 nm followed by 5 min incubation at 37°C. The FRAP level of unknown samples was evaluated using the standard curve of FeSO_4_.7H_2_O solution (0–1000 μmol/L [29]).

### Statistical analysis

The results of antioxidant assays on samples collected in PTs and GSTs were compared with those of GTs as the reference tube. For this purpose, the normality test was carried out, which showed a non-normal distribution of data, and then the Friedman test was performed. Moreover, the means and medians of MDA, NO, t-SH, and FRAP results in different groups were calculated and reported as mean ± standard error of the mean (SEM) and median (first quartile to the third quartile). The Wilcoxon matched-pairs test was used to compare mean differences between tubes. Furthermore, the Bland-Altman and Passing-Bablok regression analyses were employed to visualize the scatter of differences between glass vials and the other two collection tubes. Both the mean bias and the percentage bias were determined using the Bland-Altman analysis for medical decision points in different tubes. The evaluation of clinical significance was based on desirable bias calculation [30]. Bias was determined for each analyte in each tube as follows: Bias% = (Average absolute deviation from the target value/Target) × 100. Subsequently, the Pearson correlation coefficient (*r*) was used to determine the analyte concentrations correlation between tubes. The statistical analysis was carried out using GraphPad Prism software version 8 for Windows (GraphPad Software Inc., San Diego, CA, USA), and P-values less than 0.05 were considered statistically significant.

## Results and discussion

Preventing pre-analytical errors remains an ongoing problem in clinical and research laboratories and may affect the validity and precision of outcomes. Therefore, proper handling and usage of suitable collection tubes seem crucial. The aim of the present study was to investigate the effects of three different types of collection tubes on the measurement of MDA, FRAP, NO, and t-SH levels in order to recommend the most appropriate blood collection tube for the assessment of antioxidants. To the best of the authors’ knowledge, several studies have evaluated the effects of using various collection tubes on the alternation of chemical analytes, hormones, and drugs; however, their effect on oxidative stress factors has not been studied and it is recommended to design further studies with larger sample sizes to reevaluate these findings [8, 10, 14].

**Fig 1:**
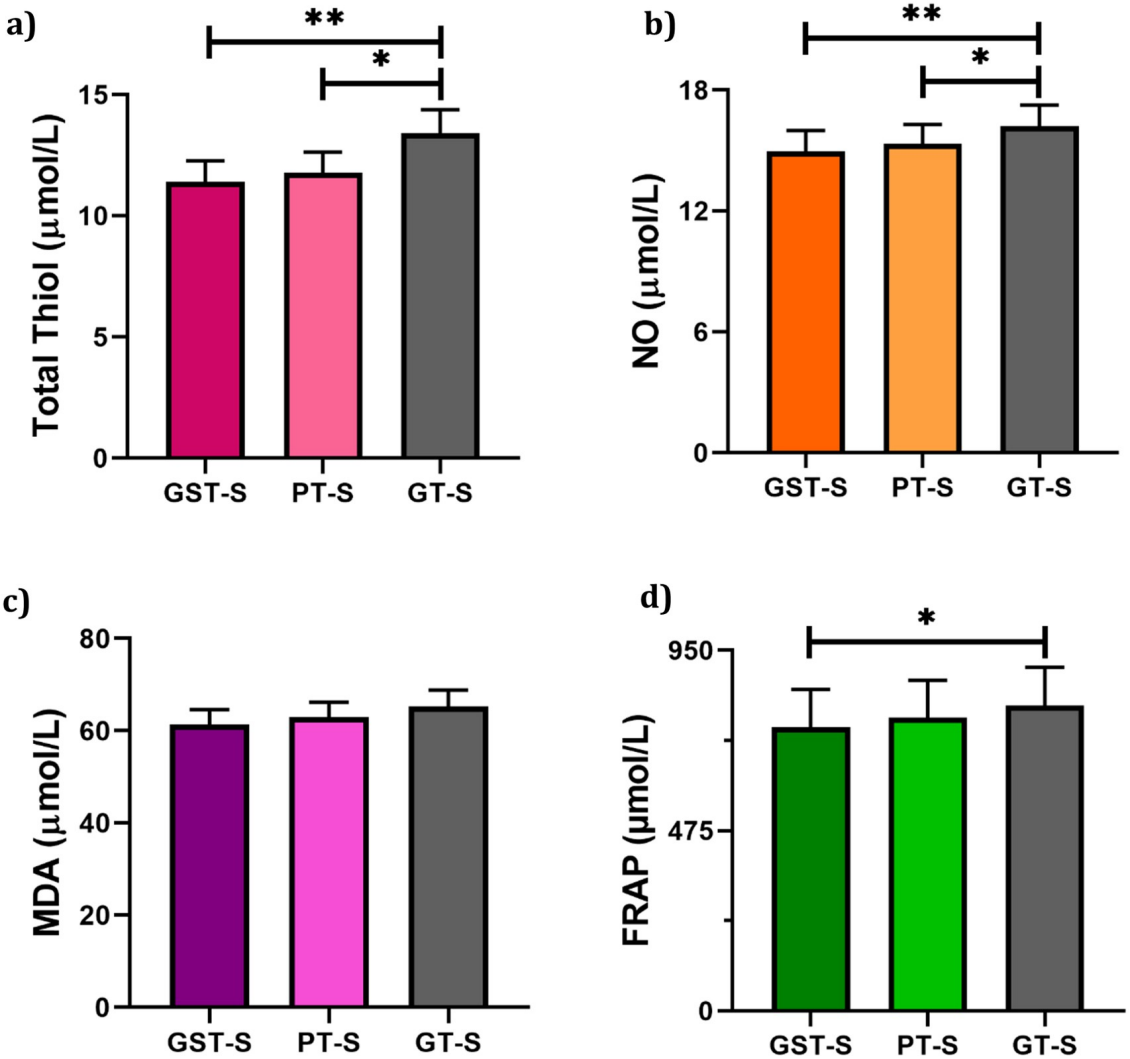
The comparison of mean concentration of (a) total Thiol, (b) NO, (c) MDA, and (d) FRAP levels in GST, GT, and PT. Wilcoxon matched paired test was used, * < 0.05, ** < 0.01.

**Fig 2:**
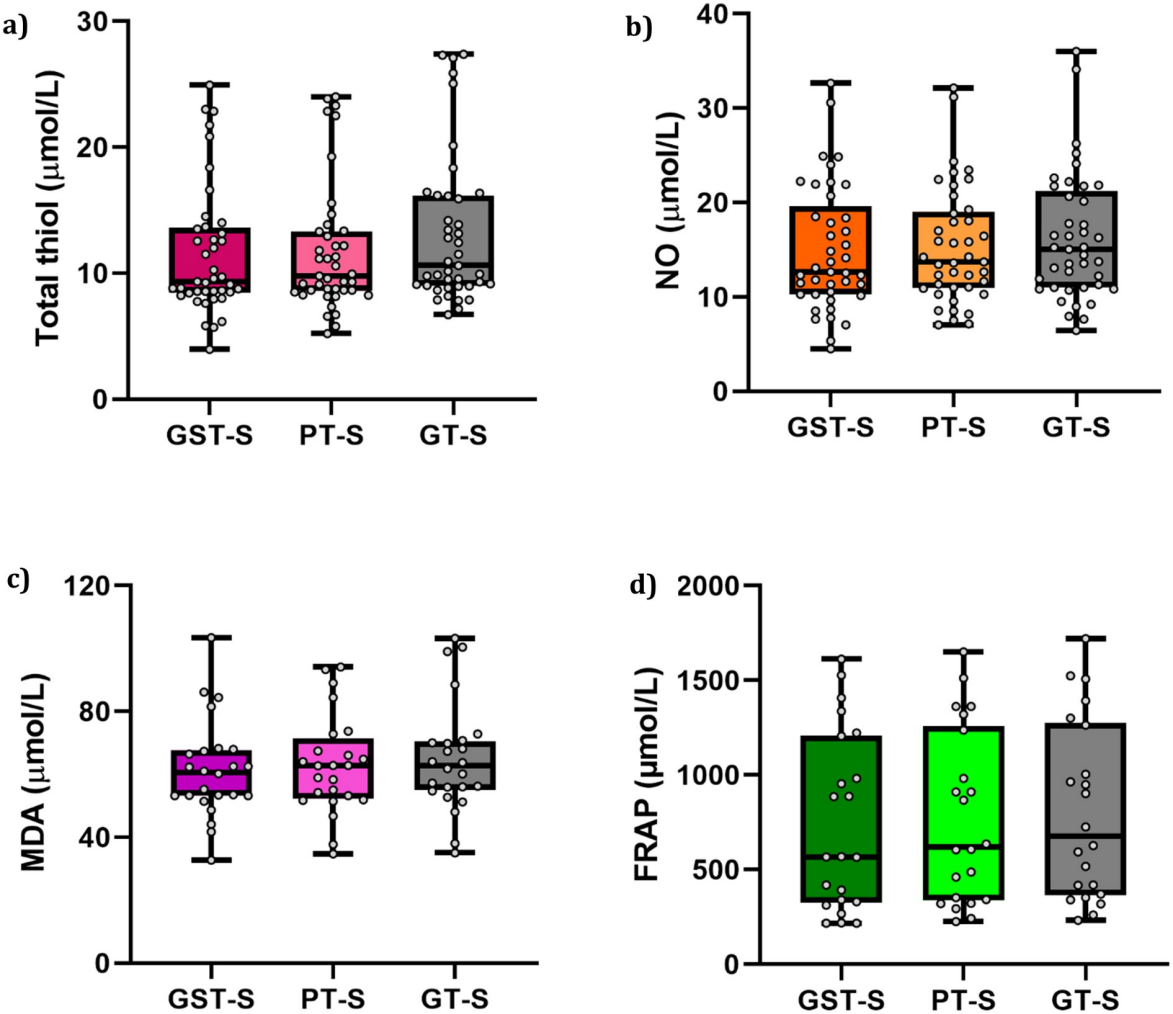
The comparison of median, mean, first, and third quartile concentration of (a) total Thiol, (b) NO, (c) MDA, *and* (d) FRAP in GST, GT, and PT.

The blood sample collected from the participants was separated into three different types of collection tubes to determine the possible impact of blood collection tube materials on serum MDA, NO, FRAP, and t-SH levels. The comparison between the means of various tubes and their significance is depicted in Fig 1. From a statistical standpoint, the findings of this study revealed that NO, FRAP, and t-SH levels were significantly lower in GSTs and/or PTs compared to GTs. We found that the t-SH content was markedly decreased in GSTs and PTs (11.42 ± 0.82, and 11.79 ± 0.83, respectively) compared to GTs (13.41 ± 0.96), and its levels were the lowest in GSTs compared to the other tubes. These data also indicated that the t-SH content in GTs showed a statistically significant difference compared to PTs and GSTs (P-value < 0.05). In addition, it was shown that the NO content was significantly lower in GSTs and PTs (14.96 ± 1.03 and 15.34 ± 0.96, respectively) than in GTs (16.20 ± 1.04). Moreover, the FRAP level was considerably lower in GSTs compared to GTs (P-value < 0.05). Based on the data obtained in this study, the MDA concentration in PTs, GTs, or GSTs did not differ significantly (P-value > 0.05). Current data suggests that PTs and GSTs may absorb or interact with these metabolites and subsequently lower their levels. It has been proposed that some tube additives such as clot activators and surfactants may absorb certain cellular materials from the whole blood specimens [8, 31]. Although we did not directly evaluate the effects of the additives in the present study, since all of the metabolites were measured in blood, this phenomenon may also apply to oxidative factors. Several studies have investigated alternations in clinical chemistry, endocrinology, serology, and molecular testing, as well as coagulation assays in PTs and GTs and have reported a statistically significant difference in some analytes [5, 32, 33]. Detailed information on the median as well as first and third quartiles of the measured factors is presented in Fig 2.

The lower concentration of factors in PTs versus GTs might be because of two reasons. The first reason is the hydrophobic surface of plastic tubes, which not only interacts with the non-polar groups of compounds but also leads to the cohesion of clotted blood on the tube wall as a result of non-smooth blood flow on the plastic surface [34, 35]. The second reason is that the clots created on the internal wall of PTs are gelatinous, which disrupts the tidy separation of clot and serum by centrifugation and correspondingly may cause hemolysis; thus, it could interfere with spectrophotometric methods employed for the assessment of antioxidants. It is also reported that clot activators fabricate a gelatinous clot and cause cell lysis as explained above [36]. To obviate this issue, it is suggested to coat the tube wall with surfactants, which are used to reduce the non-specific adsorption of red blood cells (RBCs), platelets, and proteins to the tube wall while also improving the blood flow on the tube surface [36]. These surfactants change the permeability of the cellular membrane as well as lipophilic structures due to their detergent characteristics [5]. Therefore, an increased probability of cell lysis is proposed in the case of using surfactants as an additive, which can lead to disruption in the photometric process as previously explained.

Additionally, the presence of the separator gel, which is made of viscous liquid, fillers, and/or tackifiers and can interfere with laboratory tests via several mechanisms, may explain the reduced level of factors in GSTs [1, 37]. Separator gels absorb some drugs such as phenytoin, phenobarbital, and carbamazepine through hydrophobic interactions in addition to causing a time-dependent reduction in several hormones including progesterone and testosterone [38–41]. Moreover, separator gels may also release substances such as silicone oil and gel pieces into the specimen, which would interfere with the proper function of solid-phase immunoassay systems, electrode surfaces, sample probes, and absorbance reading in the cuvettes [40, 42]. It should be noted that their rate of degradation and release may rise in the case of extreme temperatures and improper storage, which reveals the importance of suitable sample collection [40].

**Fig 3:**
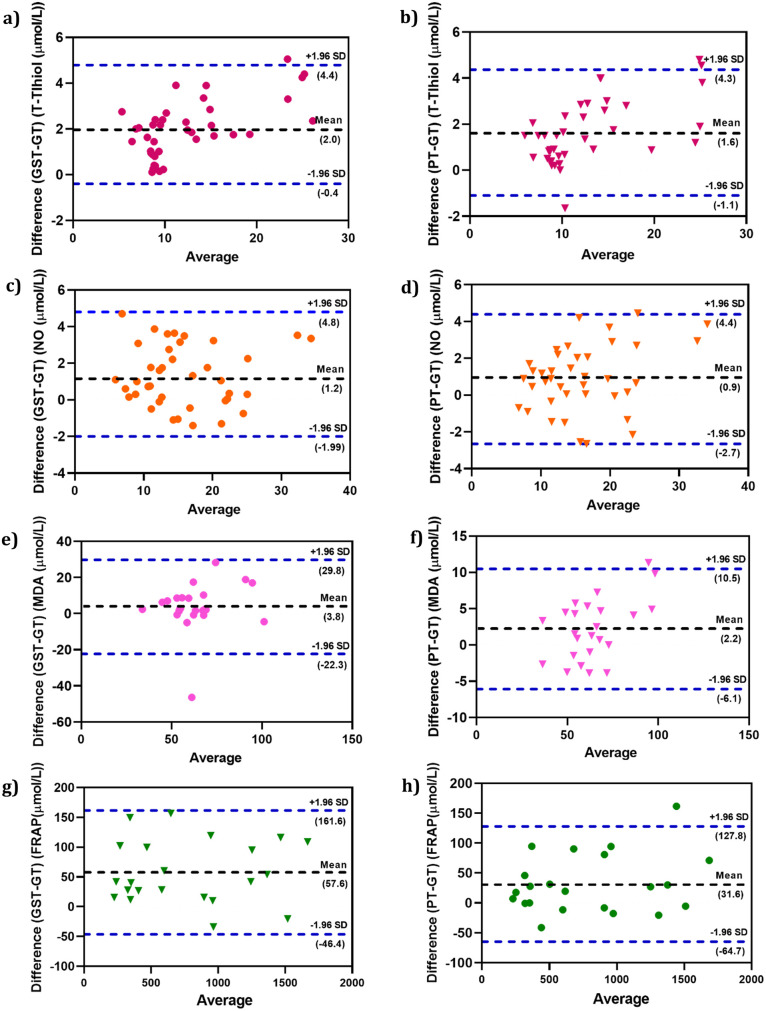
Bland-Altman difference plots for the antioxidant parameters obtained with PT, GST and GT tubes. **(a, b)** total Thiol, **(c, d)** NO, **(e, f)** MDA, **(g, h)** FRAP values. The dashed lines are the limit of agreements (LOA), which correspond to the mean ± 1.96 SD of the difference between the tubes.

**Fig 4:**
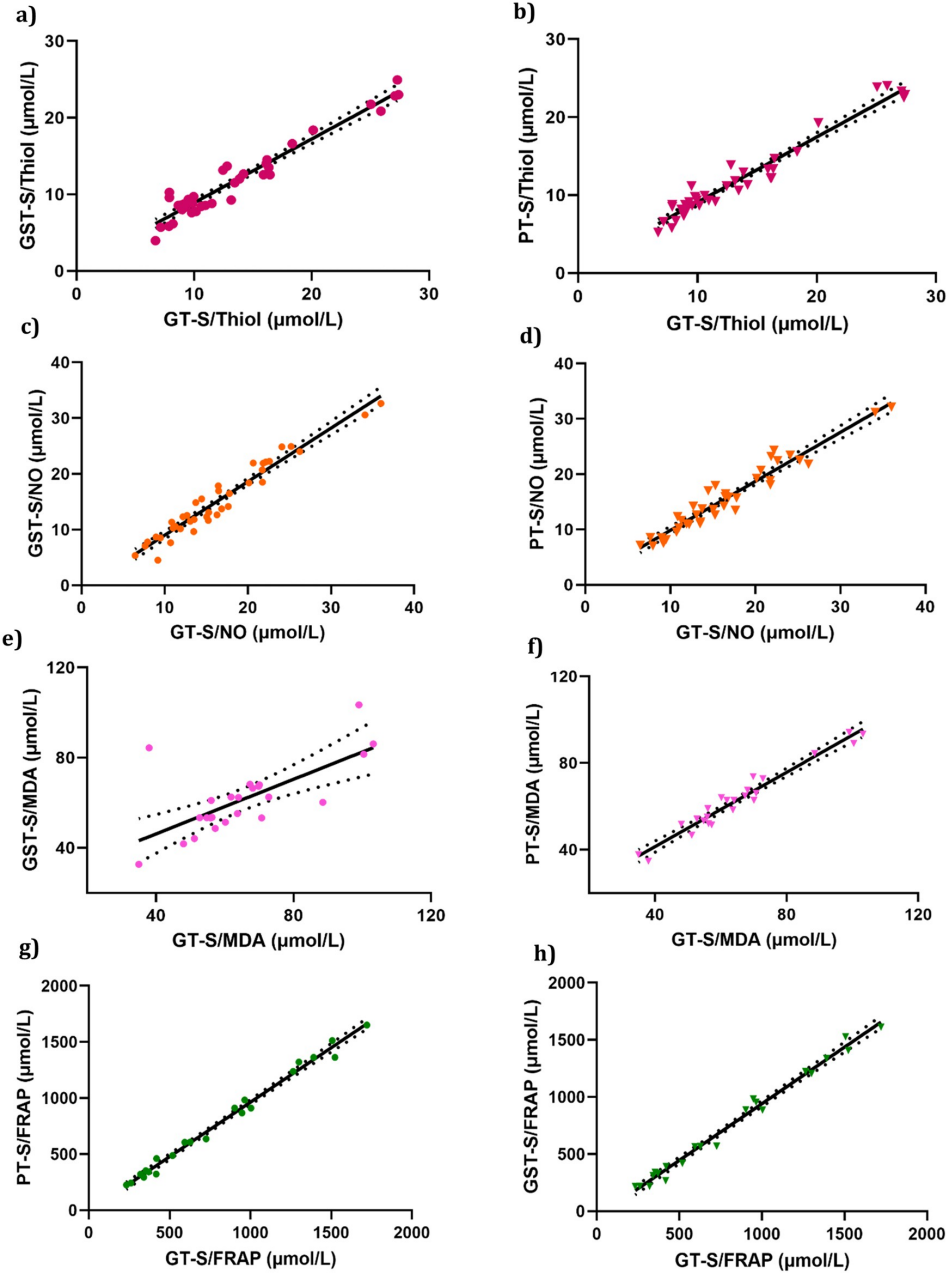
Comparison of assay results after collection of blood in PT, GT, and GST tubes, using passing and bablok regression analysis. Regression line (full line) with its 95% confidence interval (broken lines). Comparison of results for PT, GST and GT tubes for the antioxidants parameters.

**Fig 5:**
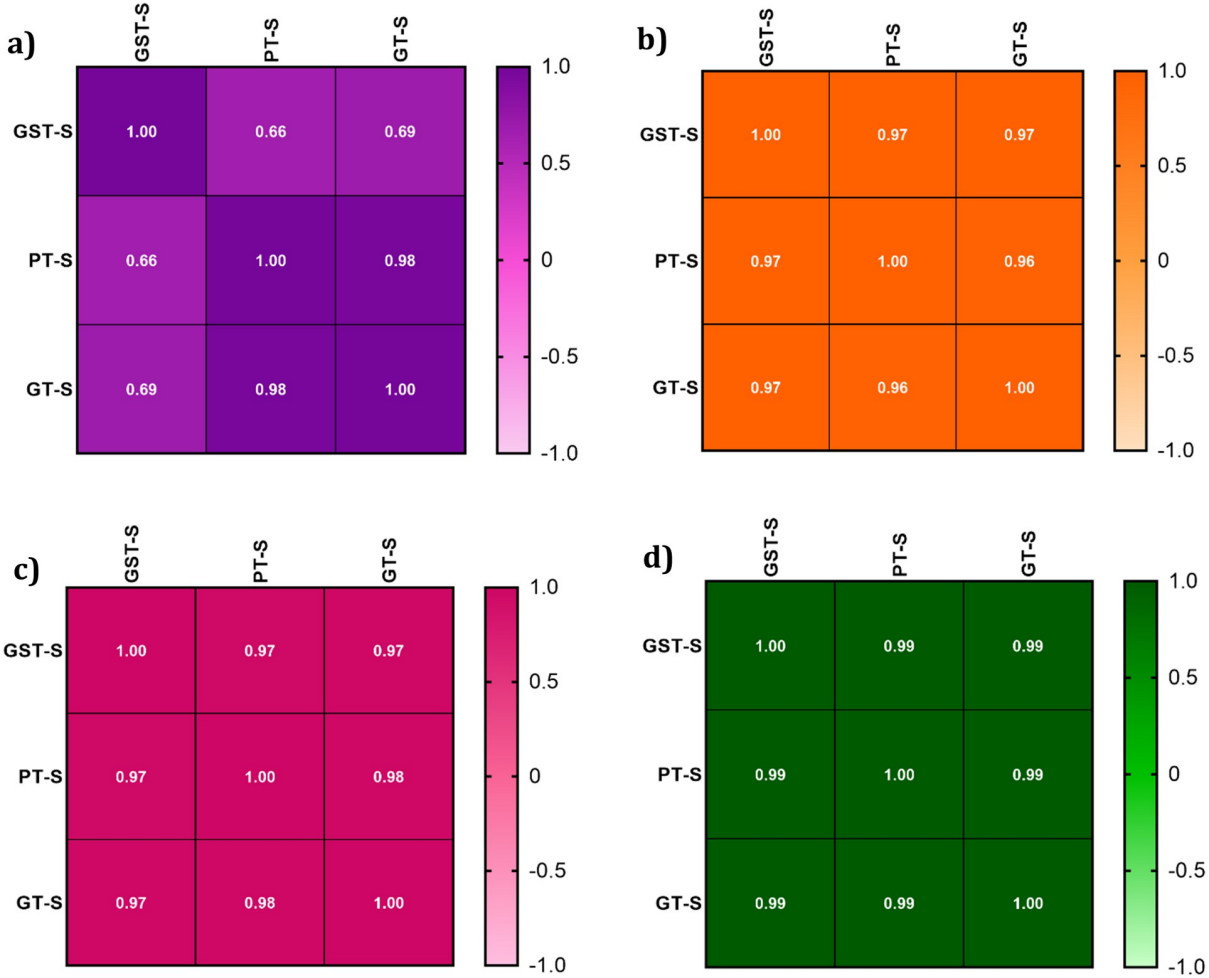
Pearson correlation coefficient (r) values of correlation analysis for the antioxidant parameters obtained with PT, GST and GT tubes. **(a)** total Thiol, **(b)** NO, **(c)** MDA, and **(d)** FRAP.

The Bland–Altman plot denoted that the different tubes had no influence on the mean NO, MDA, and t-SH values in our results (Fig 3). The mean differences (bias percentage) of NO, MDA, and t-SH ranged from 0.8% to 5.5% and were lower than the desirable calculated error or total allowable error (TAE) percentage. Variations of the obtained bias percentage are not of clinical significance. However, we found an upper 10% bias for FRAP that should be considered when using PTs and GSTs. The Passing-Bablok regression analysis (Fig 4) was performed for all the parameters. The values of the parameters in different types of tubes were strongly correlated according to the Passing-Bablok regression. In addition, Pearson correlation coefficients (r) for all tests with P < 0.05 showed a good correlation between the results in PTs vs. GTs and GSTs vs. GTs (Fig 5).

## Conclusion

Biomarkers of oxidative stress can be used in the evaluation of disease status such as cardiovascular diseases and cancer and etc. However, the use of oxidative biomarkers in prognosis of the diseases require further management. A vast number of methods have been developed and used in almost all diseases to measure the extent of oxidative stress. Nevertheless, the type of blood collection tubes has been overlooked. Based on higher Bias % in FRAP assay than TAE, it could be concluded that some of the materials used in blood collection tubes interfere with the FRAP measurement. These materials are in the tube wall and in some additives including surfactants, separating gels, and clot activators. Despite statistical differences in NO and t-SH, there was no clinically significant change between the samples. It is suggested to use GTs rather than PTs or GSTs as they show less interference in the assessment of oxidative stress biomarkers. Moreover, it is recommended to investigate the influence of different anticoagulants on the variation of these factors in prospective studies.

## Supporting information

S1 File (DOCX)
