# Supplementary material for: A comparative study on the effect of blood collection tubes on stress oxidative markers
Source: PLoS One. 2022 Apr 6;17(4):e0266567. doi: 10.1371/journal.pone.0266567 (PMC8985951; doi:10.1371/journal.pone.0266567)
Supplement: S1 File — (DOCX) [file pone.0266567.s001.docx]

**Table 1.** Data are presented as mean ± SEM concentration of MDA, FRAP, NO, and total thiol content in GST, GT, and PT.

|  | GST | PT | GT |
| --- | --- | --- | --- |
| MDA (μmol/L) | 61.46 ± 3.18 | 63.01 ± 3.19 | 65.22 ± 3.62 |
| FRAP (μmol/L) | 747.4 ± 100.30 | 773.4 ± 98.17 | 804.9 ± 100.70 |
| NO (μmol/L) | 14.96 ± 1.03 | 15.34 ± 0.96 | 16.20 ± 1.04 |
| Total Thiol (μmol/L) | 11.42 ± 0.82 | 11.79 ± 0.83 | 13.41 ± 0.96 |

**Table 2.** Data are presented as median (first quartile to the third quartile) of MDA, FRAP, NO, and total thiol in GST, GT, and PT

|  | GST | PT | GT |
| --- | --- | --- | --- |
| MDA (μmol/L) | 60.62 (53.26-67.78) | 66.79 (52.30-71.47) | 62.78 (55.08-70.58) |
| FRAP (μmol/L) | 558.10 (326.3-1210) | 621.6 (338-1258) | 676.5 (366.5-1274) |
| NO (μmol/L) | 12.64 (10.29-19.61) | 13.70 (10.96-19.06) | 15.05 (11.02-21.20) |
| Total Thiol (μmol/L) | 8.84 (8.11-13.50) | 9.79 (8.51-13.30) | 10.61 (9.04-16.20) |
